# The reliability, functional quality, understandability, and actionability of fall prevention content in YouTube: an observational study

**DOI:** 10.1186/s12877-022-03330-x

**Published:** 2022-08-09

**Authors:** Xinyi Yang, Xiaoqiang Xue, Ziqiu Shi, Sha Nan, Chengying Lian, Zhigang Ji, Yi Xie, Xiaoxuan Liu

**Affiliations:** 1 Department of Health Care, Peking Union Medical College Hospital, Chinese Academy of Medical Sciences and Peking Union Medical College, Dongcheng, Beijing, People’s Republic of China; 2 Department of Urology, Peking Union Medical College Hospital, Chinese Academy of Medical Sciences and Peking Union Medical College, Dongcheng, Beijing, People’s Republic of China; 3 Department of Gastroenterology, The 1st Affiliated Hospital of Wenzhou Medical University, Ouhai, Wenzhou, People’s Republic of China

**Keywords:** Fall prevention, YouTube, DISCERN, PEMAT, Social media, Quality

## Abstract

**Background:**

Falls are common but dangerous in the elderly. More and more seniors are searching for healthcare information online. YouTube has become the world’s most popular video streaming platform. Albeit thousands of fall prevention videos are available on YouTube, their reliability, functional quality, understandability, and actionability have not been verified.

**Methods:**

The top 300 watched videos on YouTube related to fall prevention were retrieved. After exclusion, all qualified sample videos were evaluated by three validated assessment instruments (the PEMAT scale, the HONCode scale, and the DISCERN instrument) regarding their reliability, functional quality, understandability, and actionability. Each video’s length, number of views/likes/comments, forms of expression, and the uploader’s profile were collected as well. The Wilcoxon rank sum test was performed for further analysis from the perspective of expression forms and uploaders’ identities.

**Results:**

One hundred thirty-seven videos (45.67%) were qualified as sample videos, and individuals/organizations with medical backgrounds posted 54.01% of them. Most of the excluded videos (*n* = 163) were irrelevant (*n* = 91, 55.83%), and commercial (*n* = 52, 31.90%). The median video length for sample videos was 470 seconds. The DISCERN instrument indicated that 115 videos (83.94%) were of moderate to high overall quality. Medical practitioners and organizations gained the highest scores in functional quality and reliability (*P* < 0.05), while they also tended to use technical terms more often (mean = 3.15). The HONCode scale suggested a lack of traceability was common. The most popular and actionable form of expression was workout (*n* = 58, median score = 86.90, *P* < 0.05), while monolog and keynote presentations scored the highest in understandability (no significant difference between them). The PEMAT scale suggested videos uploaded by medical teams were the easiest to be understood (*P* = 0.011 and *P* < 0.001, respectively), whereas they were less actionable than those made by fitness trainers (*P* = 0.039 and *P* < 0.001, respectively).

**Conclusions:**

Cooperation between the medical team and fitness trainers is expected for better health promotion. Plain language is advised, and sources should be provided. As for expression form, monolog or keynote presentations, plus workout clips, might be the most effective.

**Supplementary Information:**

The online version contains supplementary material available at 10.1186/s12877-022-03330-x.

## Introduction

Falls are conventional in the elderly. Studies indicated that approximately one-third of community-dwelling seniors aged 65 years or older would fall at least once a year [1, 2]. Official data revealed that unintentional falls had become the top cause of nonfatal emergency department visits of all ages in the U.S., whereas 36,508 fatal fall cases happened to seniors over 65 in 2020 [3]. Risks of falls increase with age, which would beget severe injuries and be associated with up to 12 days of delayed discharge [4–6]. Various studies and trials suggested that comprehensive interventions that addressed predisposing factors could decrease falls by approximately 25 to 80% [7–9].

The advent of the Internet has imperceptibly shifted our roles from passive knowledge recipients to active information seekers. Studies disclosed that patients dissatisfied with healthcare services were more likely to search for online health information (OHI). At the same time, internet traffic for the OHI acquisition had increased drastically from 2013 to 2018 [10, 11]. However, OHI could be either actively or passively misleading, especially for the elderly [12]. An independent study pointed out that over 40% of patients had reported a history of quitting treatment based on their online health information [13].

With a monthly active user of over 2.3 billion, YouTube has become the world’s most popular video streaming platform [14]. Official statistics showcased that the growth rate of YouTube users aged over 55 years old in the U.S. is approximately 80% higher than the overall user growth rate. Additionally, 49% of adults over 65 admitted using YouTube [15]. Notwithstanding omnifarious genres of digital content available on YouTube, its role in OHI remains inceptive.

Owing to the work of fall prevention consisting of multifactorial interventions and management, more and more seniors are searching for relevant content on the Internet. Meanwhile, many people publish fall prevention-related content on the Internet for commercial or educational purposes. To date, no research has systematically evaluated the reliability, functional quality, understandability, and actionability of fall prevention videos on YouTube. This study attempted to assess the traits mentioned above of fall prevention videos on YouTube and offer some facts-based advice on better public health engagement.

Note that since YouTube is accessible to every individual and organization, we here defined the “quality” we would discuss later as functional quality. On the other hand, technical quality, including video and audio signals, color and resolution, visual effects and postediting techniques, etc., would not be discussed due to variation in filming conditions and equipment. Functional quality is all about the usefulness of the uploaded content, such as the comprehensiveness and guiding value of treatments, management, suggestions, and recommendations presented in the sample video and its role in promoting health and preventing falls. Reliability refers to the extent viewers could trust the sample video. A less reliable video may contain unfair conjecture, disinformation, misinformation, or content that could not be sourced. Good understandability indicates materials are understandable when consumers of diverse backgrounds and varying levels of health literacy can process and explain key messages. Good actionability refers to the patient education materials being actionable when consumers of diverse backgrounds and different levels of health literacy can identify what they can do based on the information presented.

## Methods

### Ethics approval

The principal investigator’s hospital’s Institutional Review Board approved the study verbally. All methods were carried out in accordance with relevant guidelines and regulations of the hospital. This study method had been proven harmless in previous studies. Waiver of documentation of informed consent was permitted because this study conducted no experiments on humans, tissue samples, or data. No patient’s privacy was disclosed in sample videos.

### Search strategy and samples processing

**Fig. 1:**
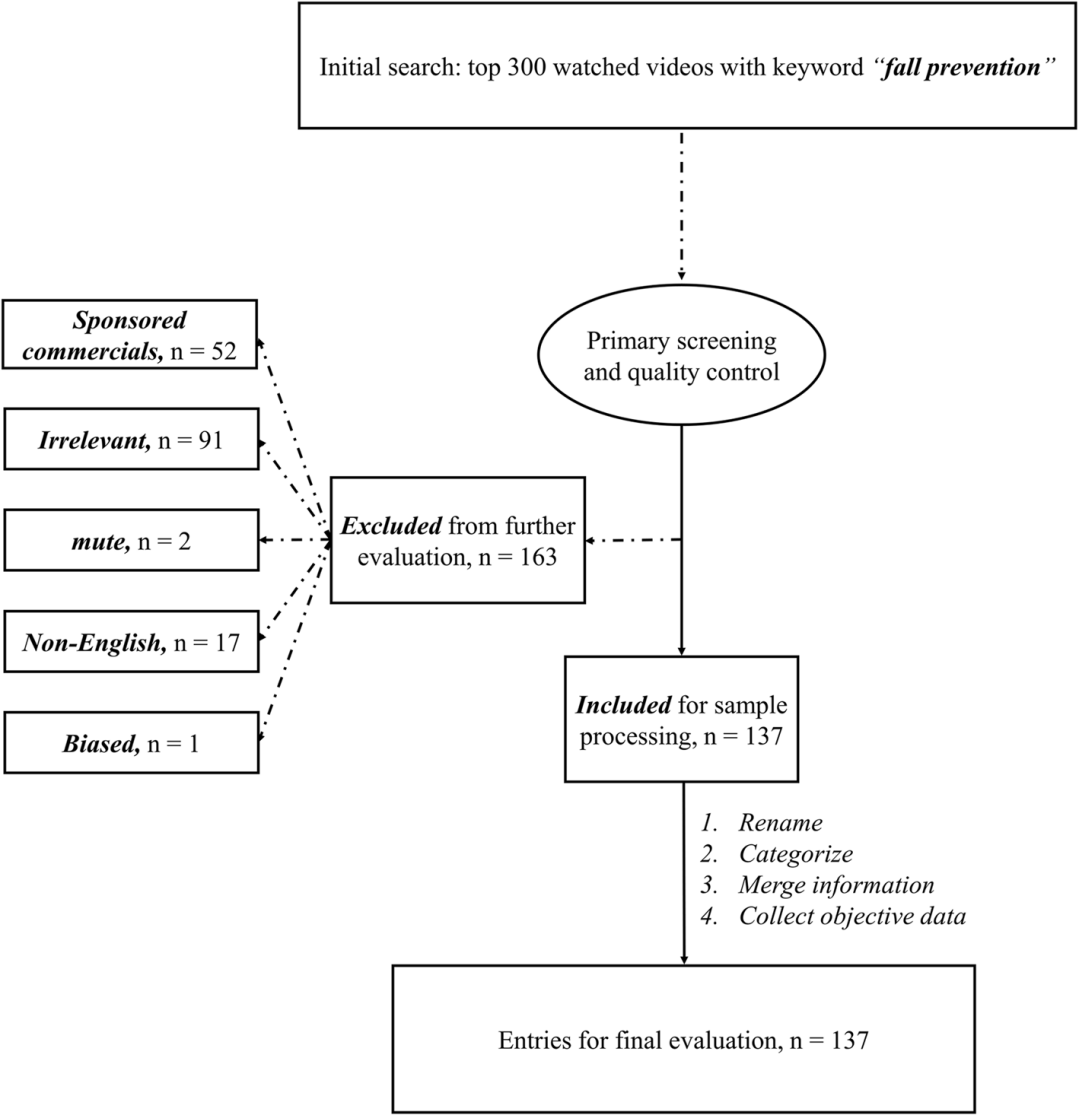
The detailed inclusion and exclusion flowchart

A Python script was written to retrieve the most-watched 300 YouTube videos on “fall prevention” by 25th April 2022. Descriptive information was collected as well, including A) the length and description of each video, B) numbers of views/likes/dislikes, and comments, C) the date when the video was uploaded, D) forms of expression, namely the genre of the video and how it was presented, including videos in the format of workout, introduction and demonstration, cartoon, monolog, keynote presentation, and drama/skit, and E) the uploader’s profile. All videos should be presented in English. Further evaluations were made to exclude videos with irrelevant contents (prevention for construction fall, hair fall, etc.), obvious commercial promotions (fall prevention sneakers, socks, etc.), potential copyright disputes, and linguistic barriers (mute, Hindi, etc.). The detailed inclusion and exclusion flowchart can be tracked in Fig. 1.

Note that this research was designed to analyze the quality of OHI for the public. Hence, videos intended for medical professionals, such as webinars, lectures, and video conferences, were also excluded. After preliminary screening, 137 videos were regarded as sample videos. Sample videos were renamed and de-identified before being sent for review.

### Evaluating methodologies

The DISCERN instrument was first published in 1999 by the Division of Public Health and Primary Care at Oxford University, London, to rate the quality of written information regarding treatment choices for health problems [16]. The instrument is made by 15 questions plus an overall quality rating (Question 16). Each question is rated from 1 to 5 points. Its robustness is still reliable today, inasmuch as it has been widely applied in gauging OHI on YouTube and TikTok [17, 18]. The reliability of the OHI was evaluated by questions one to eight of the DISCERN, and questions nine to fifteen were used to assess its functional quality.

The Health on the Net Code (HONCode) is a code of conduct comprising eight procedural principles that attempt to assist customers in identifying the understandability, accessibility, and credibility of OHI [19].

The Patient Education Materials Assessment Tool (PEMAT) is a coded scale that reflects the understandability and actionability of a sample video [20]. It is currently the most frequently used scale for assessing patient education materials. The weight and score of each question could be automatically calculated by using the auto-scoring form provided by the PEMAT team [21].

Detailed HONCode scoring criteria and the DISCERN instrument are available as supplementary materials (Additional file 1: Appendix A, Additional file 2: Appendix B, and Additional file 3: Appendix C). The PEMAT auto-scoring form can be accessed online [21]. Two raters who were previously blinded from the preliminary screening were responsible for the independent quality evaluations. Cohen’s kappa coefficient was calculated to measure the degree of inter-rater reliability. Verdicts were calculated as mean scores by another author. All videos could be accelerated but cannot be skipped during the review.

### Data processing and analysis

Descriptive information was collected for all 137 sample videos, including video length, description, date of the post, and numbers of views/likes/dislikes/comments. We also categorized all types of videos into two dimensions based on the forms of expression and the uploader’s profile. By the end of the data processing, there were five types of the identities for uploaders: 1) fitness trainers, 2) medical professionals: nurses, physiotherapists, geriatricians, etc. 3) non-professional individuals, 4) professional organizations: medical centers, centers for disease control and prevention, official county channels. And 5) non-professional organizations. As for forms of expressions, the number of categories was six: 1) workout, 2) introduction and demonstration, 3) cartoon, 4) monolog, 5) keynote presentation and 6) drama/skit. We aim to analyze the potential significant difference between different types of YouTubers and forms of expression. Hence, C (5,2) = 10 pairs of uploaders combinations and C (6,2) = 15 pairs of forms combinations.

Six different scores would be calculated based on the DISCERN instrument and PEMTA scale: the overall quality score (DISCERN instrument), the reliability score (DISCERN instrument), the functional quality score (DISCERN instrument), the total score (DISCERN instrument), the understandability score (PEMAT scale), and the actionability score (PEMAT scale).

Statistical Product and Service Solutions version 24.0 (SPSS, IBM Corp.) was used for data analysis. The measurement data of normal distribution was described by “range (mean ± standard deviation)”; the measurement data of skew distribution was characterized by “median (interquartile range).” The Wilcoxon rank sum test was performed for deeper analysis between pairs. The level of significance was set at 0.05.

## Results

### Description of sample characteristics

The preparative appraisal excluded approximately half of the retrieved videos (*n* = 163, 54.33%) as they were perceived as irrelevant (*n* = 91, 55.83%), promotional (*n* = 52, 31.90%), non-English (*n* = 17, 10.43%), mute (*n* = 2, 1.23%) or biased (n = 1, 0.73%). The rest (*n* = 137, 45.67%) were regarded as sample videos. The kappa coefficient was 0.821, indicating there was a strong consistency between results from the two raters.

All sample videos attempted to enhance viewers’ awareness of falls through patient education. A vast majority of them also delivered in-home exercise content, be it the OTAGO exercise, Tai Chi, yoga, etc. It was no surprise to know that professional organizations contributed the most (*n* = 44, 32.12%), followed by fitness trainers (*n* = 32, 23.36%) and medical professionals (*n* = 30, 21.90%). In video length, the median number was 470 seconds. Professional organizations posted the shortest (309 seconds, 90–3699 seconds) while fitness trainers published the longest (942 seconds, 63–4795 seconds). Surprisingly, although non-professional individuals uploaded the least number of videos (*n* = 14, 10.22%), their posts were the most liked (39.5 likes, 0–5736 likes) and second most-watched (1708 times, 95–60,482 times). The median time duration from publishing to 25th April 2022 for all sample videos was 756 days (21–4883 days). No relationship between time of publishing with view count was found.

In terms of expression forms, workout was adopted by the most (*n* = 58, 42.34%), followed by introduction and demonstration (*n* = 33, 24.09%), monolog (*n* = 19, 13.87%), keynote presentation (n = 14, 10.22%), drama/skit (*n* = 11, 8.03%), and cartoon (*n* = 2, 1.46%).

When we put dimensions of uploader’s identity and some of the most popular expression forms combinedly, more than half of workout videos (*n* = 32, 55.17%) were made by fitness trainers, followed by medical professionals (*n* = 8, 13.19%), non-professional organizations (n = 8, 13.19%), professional organizations (*n* = 6, 10.34%), and non-professional individuals (*n* = 4, 6.90%). In addition, 28 out of all 33 introduction and demonstration videos (84.85%), 15 out of all 17 monolog videos (88.24%), and 10 out of all 14 keynote presentation videos (71.43%) were posted by medical professionals and professional organizations. Detailed characteristics of all sample videos are presented in Table 1.

**Table 1:** General characteristics of all evaluated videos

Categories	Views, (m, IQR)	Length, s, (m, IQR)	Likes, (m, IQR)	Comments, (m, IQR)
**Identities (n, %)**				
Fitness trainers (32, 23.36%)	1546.5 (303.75–3942.5)	942 (340–2563.75)	25 (2–90.75)	1 (0–8.5)
Medical professionals (30, 21.90%)	1317.5 (381–23,411.25)	486 (263.25–1298.25)	12 (2.25–233)	1 (0–9.75)
Non-professional individuals (14, 10.22%)	1708 (210.75–7696.75)	509.5 (239.75–1057)	39.5 (4–113.75)	3 (0–13.25)
Professional organizations (44, 32.12%)	3203.5 (384–10,811.25)	309 (183.75–624.25)	19.5 (4–74)	0 (0–2)
Non-professional organizations (17, 12.41%)	203 (143–501)	311 (175–590)	3 (2–5)	0 (0–0)
**Forms of expressions (n, %)**				
Workout (58, 42.34%)	1168 (321.25–6140)	658.5 (314.25–1979.5)	19.5 (3.25–115.5)	1 (0–7.5)
Introduction and demonstration (33, 24.09%)	1396 (309–5070)	311 (190–470)	5 (0–30)	0 (0–1)
Cartoon (2, 1.46%)	154,133.5 (109,661.25-198,605.75)	168 (134–202)	1256 (753–1759)	23 (18–28)
Monolog (19, 13.87%)	379 (154.5–7301.5)	232 (118–1100.5)	9 (1–27)	0 (0–2.5)
Keynote presentation (14, 10.22%)	247 (133.5–2677.75)	542.5 (311.25–1011.25)	3 (1.25–17.75)	0 (0–1)
Drama/skit (11, 8.03%)	11,472 (2692.5-25,030.5)	612 (309–990.5)	95 (19.5–129.5)	5 (1.5–7)

*M* Median, *IQR* Interquartile range, *S* Seconds, *N* Number

### The reliability, functional quality, understandability, and actionability of sample videos

Results from the HONCode scale suggested the most often ignored principle when publishing OHI was the traceability of the information. Namely, any statement published should be provided with references or hyperlinks. This common neglect was slightly better in videos shot by medical professionals and professional organizations. At the same time, the least addressed was in videos made by fitness trainers and non-professional individuals/organizations. However, no statistical significance was found between all five types of YouTubers. In the form of expression, the HONCode scale indicated that the workout gained the fewest points in terms of traceability, as most of the videos were simply full-length workout recordings. Detailed results of the HONCode scale can be found in Additional file 4.

Four sets of scores were calculated from the DISCERN instrument, and two were from the PEMAT scale. In short, medical professionals and professional organizations scored quantitatively the highest in the total DISCERN scores (40.10 ± 6.46 versus 40.39 ± 5.55, respectively). The result was in accordance with the overall quality scores (Question 16, points: 3.73 ± 0.56 versus 3.80 ± 0.57, respectively). In the overall quality section of the DISCERN instrument, 115 out 137 videos (83.94%) scored three points or above, indicating that 83.94% of sample videos were of moderate to high quality.

To dive deeper, we calculated two sub-scales and noticed that medical professionals and professional organizations scored quantitatively the highest in both reliability (22.18 ± 4.23 versus 22.23 ± 2.98, respectively) and functional quality (17.92 ± 3.22 versus 18.16 ± 3.24, respectively). A mosaic plot is presented in Additional file 5 for visualizing the results of the DISCERN instrument. In that plot, each cell represents a particular given point (e.g., blue for one point and red for five points). By looking at the approximate distribution and proportion of those colorful bricks, we could grasp a general idea of the distribution and proportion of the scores from the DISCERN instrument. For instance, videos made by non-professional individuals and fitness trainers generally scored low (in blue color) in the “source” and “risk” related questions. In contrast, this situation would be changed if videos were posted by YouTubers with medical backgrounds. Also, videos in the form of workouts gained more one-point (blue mosaics) than that in other forms.

In the PEMAT scale, the first two with the highest scores in the understandability aspect were still medical professionals and professional organizations (80.90 ± 12.23 versus 82.68 ± 9.37, respectively). Fitness trainers scored the highest in actionability (87.63 ± 15.98). Non-professional individuals scored the lowest on the DISCERN scale and the PEMAT scale. Detailed scores have been listed in Table 2.

**Table 2:** Six sets of scores for different identities and forms of expressions

Categories	DISCERN-Overall quality	DISCERN-reliability	DISCERN- Functional quality	DISCERN-Total score	PEMAT-Understandability	PEMAT-Actionability
**Identities (n)**	**Mean ± S.D.**	**Mean ± S.D.**	**Mean ± S.D.**	**Mean ± S.D.**	**Mean ± S.D.**	**Mean ± S.D.**
Fitness trainers (32)	3.16 ± 0.54	17.88 ± 1.71	14.78 ± 2.68	32.66 ± 3.41	70.63 ± 15.12	87.63 ± 15.98
Medical professionals (30)	3.73 ± 0.56	22.18 ± 4.23	17.92 ± 3.22	40.10 ± 6.46	80.90 ± 12.23	77.10 ± 19.40
Non-professional individuals (14)	2.64 ± 0.67	15.79 ± 2.83	12.82 ± 2.40	28.61 ± 4.22	64.21 ± 18.30	59.57 ± 22.65
Professional organizations (44)	3.80 ± 0.57	22.23 ± 2.98	18.16 ± 3.24	40.39 ± 5.55	82.68 ± 9.37	76.18 ± 14.63
Non-professional organizations (17)	3.47 ± 0.47	17.59 ± 2.00	16.12 ± 1.68	33.71 ± 1.90	71.65 ± 11.08	66.94 ± 11.49
**Forms of expressions (n)**	**Mean ± S.D.**	**Mean ± S.D.**	**Mean ± S.D.**	**Mean ± S.D.**	**Mean ± S.D.**	**Mean ± S.D.**
Workout (58)	3.24 ± 0.66	18.28 ± 2.84	14.81 ± 2.92	33.09 ± 4.73	71.16 ± 14.55	86.90 ± 17.43
Introduction and demonstration (33)	3.77 ± 0.60	21.61 ± 3.96	18.65 ± 3.00	40.26 ± 6.40	78.85 ± 11.82	73.97 ± 15.83
Cartoon (2)	3.75 ± 0.75	18.00 ± 3.50	16.50 ± 3.50	34.50 ± 7.00	76.50 ± 0.00	67.00 ± 4.00
Monolog (19)	3.32 ± 0.59	19.53 ± 3.16	16.18 ± 3.07	35.71 ± 5.02	81.42 ± 11.90	63.42 ± 10.43
Keynote presentation (14)	3.93 ± 0.53	23.21 ± 4.12	18.79 ± 3.00	42.00 ± 6.57	83.50 ± 11.20	67.43 ± 18.03
Drama/skit (11)	3.55 ± 0.54	20.27 ± 4.32	17.18 ± 2.50	37.45 ± 6.10	74.09 ± 17.86	60.82 ± 13.11

*N* Number, *S.D.* Standard deviation

The Wilcoxon rank sum test analyzed both scales’ significance between identity-identity and form-form pairs. Results of 150-pair calculations suggested that no significant difference had been found regarding the reliability, functional quality, understandability, and actionability of fall prevention videos on YouTube between medical professionals and professional organizations (*P* = 0.541, 0.604, 0.407, and 0.630, respectively). In contrast, medical professionals and professional organizations uploaded videos were the most reliable, and non-professional individuals were the least reliable. Although medical professionals and professional organizations outperformed fitness trainers on the scale of understandability (*P* = 0.011 and *P* < 0.001, respectively), their videos were less actionable than those produced by fitness trainers (*P* = 0.039 and *P* < 0.001, respectively). No significant difference had been found between non-professional individuals and non-professional organizations in terms of actionability (*P* = 0.242).

The form of workout gained the best actionability (86.90 ± 17.43, significance was found between it and any other form), whereas the worst understandability (71.16 ± 14.55, significance was found between it and any other form). However, no significance was found between the monolog and keynote presentation in terms of understandability (*P* = 0.427). In the aspect of reliability, keynote presentation scored the highest (23.21 ± 4.12), while introduction and demonstration scored the second (21.61 ± 3.96), whereas no statistical significance was found (*P* = 0.200). The same story could be told in functional quality and total DISCREN (*P* = 0.692 and 0.463, respectively). Detailed results of the Wilcoxon rank sum tests could be found as supplementary materials (Additional file 6: Appendix D).

## Discussion

Walking is an activity that we almost take for granted: just put one foot in front of the other and propel ourselves somewhere. It could be with a purpose or just for fun. However, just as lightning and storms often occur together, walking and potential falling usually accompany. According to the official data released by the U.S. CDC, unintentional falls have become the leading cause of nonfatal emergency department visits of all ages in America [3]. Worse, the risks of falls increase with age [4, 6]. Roughly every one in three Americans aged 65 years or older would experience at least one fall annually [1]. A variety of risk factors, such as unsafe or unfamiliar environment, poor vision, weakness, unsteady gait, loss of muscle strength, prescriptions of certain medications, mental confusion, and dementia, are associated with falls in the elderly [22, 23]. Fall prevention is a comprehensive measure that consists of risk assessment and stratification, creating a safe environment, education, exercise, vision examination, prescription checking, and treating primary diseases [8, 23].

The popularization of the Internet and mobile networking has progressively catalyzed social media platforms into banks of online health information for the broad public. Song et al. suggested people in some countries and regions showed more trust in experience-based health information sources (e.g., blogs, online support groups, social networking sites) than people in other places [24]. As video is naturally more intuitive and vivid than the textbook, we could foresee the general public turning to video tutorials for help when faced with a conundrum.

However, publishing OHI videos could be causal and misleading due to its low entry threshold. Previous studies suggested that approximately 60–80% of OHI on YouTube was less satisfying in quality, and nearly 10–30% of them could be misleading or biased [17, 25, 26]. As the seniors are more vulnerable to low-quality OHI, and the misleading or erroneous OHI is more possibly to be relayed amongst the elderly [12, 27], it is high time for us to examine online healthcare videos they are exposed to and maybe, produce model videos that meet their needs. This study was designed to evaluate fall prevention videos’ reliability, functional quality, understandability, and actionability on YouTube and provide our advice on better public health promotion.

### Major findings

The results of the searched videos on YouTube were complicated. Most of the excluded videos (*n* = 163) were irrelevant (*n* = 91, 55.83%), and commercial (*n* = 52, 31.90%). The irrelevant ones could be found at a single glance as they were intended for “scaffolding fall prevention,” “hair fall prevention,” or “winter sports fall prevention.” However, for videos containing potential promotional content, sometimes even the author could not recognize it immediately. For some videos, further discussions with another author with medical background before making decisions were required.

After preliminary screening of all videos, the YouTube homepage was flooded with continuous flows of advertisements and similar recommendations due to the big data algorithm. Most of them had captivating titles and enjoyed many likes and views. Whether the algorithm would cause secondary dissemination of irrelevant or even misleading information is worth studying. Nevertheless, we must admit some commercial content is not directly delivered in traditional advertising, but the form of reviews, shares, and recommendations. We were concerned that some implicit commercial content on YouTube might be complex for older people to distinguish, as sometimes we had a hard time identifying potential promotions.

One hundred fifteen out of 137 videos (83.94%) scored three points or above overall quality. Namely, nearly 85% of sample videos were of moderate to high overall quality. This result seemed to contradict formally published data that rates of videos of moderate to high quality were below 40 [17, 28]. However, we believe two possible reasons might account for it. First, when we compared the result with videos addressing benign ailments, such as postoperative pelvic floor exercises [29], the rate was comparable (83.94% versus 70.6%). Second, as some commercials were so ambiguous that most elderly would not notice, they could get more searching results, and the rate would be lowered a little.

Due to the comprehensiveness of fall and fall prevention, illustrating its risk factors and targeting interventions thoroughly within a median video length of 470 seconds seemed to abridge some details inevitably. On the other hand, as older people’s attention, stamina, and vigor would decrease with age [30], videos with long lengths might hinder audiences’ concentration. Therefore, we assumed one of the key points to improving video quality was how to deliver the most helpful information in a limited time. To escape this predicament and increase people’s understanding of OHI, some scholars advised medical professionals to be more engaged in the video production process [18, 25]. It is conceived that they should have more contact with the elderly in their daily work and could make choices between information [31].

In this study, we noticed that more than half of the sample videos were uploaded by individuals/organizations with medical backgrounds (*n* = 74, 54.01%). As it turned out, they did score statistically the highest in the understandability on the PEMAT scale (*P* < 0.05). From the form perspective, monolog and keynote presentations scored the highest as they were commonly rich in pictures, flash, and outlines. We believe videos with rich audio and detailed outlines should be the easiest to understand.

Individuals/organizations with medical backgrounds also gained the highest functional quality and reliability (*P* < 0.05). Potential explanations were: first, topics in their videos were relatively more straightforward (see there were more red and orange bricks in those two groups upon Question 1–3 in Additional file 5 indicating that they scored higher in Question 1–3); second, traceability was more evident in their videos (notice there were fewer blue and artichoke green bricks in those two groups upon Question 4–7 in Additional file 5). Honestly, a more straightforward opening could give the audience a more precise thought on what they need to do and how to do it and save time.

This study also found that 10.95% of videos on YouTube had at least one unexplained medical term, such as hypostatic pneumonia and hallux valgus deformity. Professional individuals and organizations tended to use technical terms more often (mean = 3.15), although this did not affect their scores in understandability. This result is lower than previous studies on genitourinary tumor content on YouTube and TikTok [17, 18, 28]. One reason might be the difference in median video length (470 seconds versus 273 seconds versus 43 seconds, respectively). Also, content on fall prevention was less sophisticated than tumor treatment. Therefore, YouTubers here should have sufficient time to define terms. Overall, health promotion should benefit from adopting plain language and well-prepared paperwork.

We, as medical practitioners, were encouraged to find more and more professional organizations setting up official accounts and publishing professional content on social media during the appraisal process. On the one hand, we also call on medical professionals to introduce themselves and improve the traceability of the video by posting sources actively on screen or in the video description below. As the HONCode suggested, the traceability of their videos was not significantly better. On the other hand, cooperation between medical professionals and non-medical parties is expected. Because there were some “not applicable” answers in the PEMAT scale, visualizing it as a mosaic plot was inaccessible.

In actionability, we noticed that fitness trainers scored the highest from the perspective of the uploader’s identity, while from the standpoint of the form of expression, workouts gained the highest. Since all fitness trainers had contributed to workout videos (*n* = 32, 55.17%), we believe that high scores from workout videos were associated with fitness trainers’ expertise in exercise curriculum. However, we also noticed that the videos released by fitness trainers had the most extended median video length meanwhile the least verbal communication: most of them were simply in an “exercise with me” format. This might partly explain why workout scored the least on the HONCode scale.

Also, difficulty ratings, alternatives, playbacks, and other relevant background information were lacking. This should explain why they ranked bottom in understandability. As some workout sections were presented in cartoon and keynote, no significant difference was found between the form of introduction and demonstration, cartoon, and keynote presentation.

Hence, we would like to suggest: first, more background information and relevant explanations should be given before and during the exercise, by which the traceability and understandability could be improved; second, the entire exercise length should be shortened or the training should be divided into several sections; third, a difficulty rating system should be established as some videos were too intense in training for the elderly. We also look forward to seeing the cooperation between medical teams and fitness trainers.

### Expectations and limitations

The role of YouTube in engaging public Health has dramatically grown during the COVID-19 pandemic [32, 33]. Due to its broad user base and fast-food-style video streaming algorithm, YouTube has become a place of strategic importance in the battle for digital health. Based on the status, functional quality, reliability, understandability, and actionability of fall prevention videos on YouTube, we appeal for more interaction between us as health care practitioners and the Internet.

Cooperation between the medical team and fitness trainers is expected for better health promotion. Monolog or keynote presentations, plus workout clips, are supposed to be the most effective. Online videos should be rich in audio and plain language in understandability. Sources should be provided, and a rating system for intervention should be established. As the Internet has a wide range of radiation, one action might quickly help thousands of users, young or old, at the same time.

Several limitations should be noted in this article. First, all videos were in English. High-quality videos in other languages might be omitted. Second, we did not study the relationship between video comments and misleading information due to the limited time. Some had put that there would be some advertisements or deceptive information in the comment sections below YouTube videos [28].

Based on this study, we suggest a few directions for future research. Future research should focus more on video content, such as misleading information, outdated interventions, etc. In addition, if possible, the elderly should speak for themselves and audit the quality of the video.

## Conclusion

Most fall prevention videos on YouTube are of moderate to good overall quality. However, their reliability, functional quality, understandability, and actionability varied from uploader to uploader, form to form. Actions are needed to make online healthcare content better.

## Supplementary Information

**Additional file 1.** 

**Additional file 2.** 

**Additional file 3.** 

**Additional file 4.** 

**Additional file 5.** 

**Additional file 6.** 

## Data Availability

The original data that support the findings of this study are available on request from the corresponding author [YX].

## Abbreviations

OHI: Online health information
HONCode: The Health on the Net Code
PEMAT: Patient education materials assessment tool
