# Supplementary material for: The reliability, functional quality, understandability, and actionability of fall prevention content in YouTube: an observational study
Source: BMC Geriatr. 2022 Aug 9;22:654. doi: 10.1186/s12877-022-03330-x (PMC9362965; doi:10.1186/s12877-022-03330-x)
Supplement: Supplementary file 1 — Additional file 1. [file 12877_2022_3330_MOESM1_ESM.pdf]

The Health on the Net Code (HONCode) Principles and Its Results

HONCode Principles	Number of videos in compliance
1. Any medical or health advice given in the video must come from a qualified health professional unless it is clearly stated that the information does not come from a qualified health source.	N = 120 (87.59%)
2. The information provided in the video must be designed to support the patient's self-management, but it is not meant to replace the patient-physician relationship.	N= 106 (77.37%)
3. The information in the video maintains the right to confidentiality and respect of the individual patient featured.	N = 125 (91.24%)
4. Each video contains references to source data on information presented or contains a specific HTML link to source information.	N = 64 (46.72%)
5. Each video containing claims on the benefits or performance of specific skills/behaviors, interventions, treatments, products, etc., must be supported by evidence through references or HTML links.	N = 40 (29.20%)
6. The video must provide the viewer with contact information, or a URL to more information.	N = 136 (99.27%)
7. Any individual or organization that contributes funds, services or material in the posted video must be clearly identified in the video or video description.	N = 133 (97.08%)
8. If advertisement supports funding to the video or the video's developers, it must be clearly stated. Included advertising must be clearly differentiable to the viewer: There should be a clear difference between the advertising material and the educational material in the video.	N = 18 (13.14%)

Data are shown as number (percent).
Scoring: Low = satisfying 0 to 2 principles; Medium = satisfying 3 to 5 principles; High = satisfying 6 to 8 principles.
Abbreviations: HTML = hypertext markup language; URL = uniform resource locator.
