# Supplementary material for: The reliability, functional quality, understandability, and actionability of fall prevention content in YouTube: an observational study
Source: BMC Geriatr. 2022 Aug 9;22:654. doi: 10.1186/s12877-022-03330-x (PMC9362965; doi:10.1186/s12877-022-03330-x)
Supplement: Supplementary file 2 — Additional file 2. [file 12877_2022_3330_MOESM2_ESM.pdf]

The DISCERN questionnaire

Section 1 – Is the publication reliable?	
1. Are the aims clear	No Partially Yes<br>1 2 3 4 5
2. Does it achieve its aims?	No Partially Yes<br>1 2 3 4 5
3. Is it relevant?	No Partially Yes<br>1 2 3 4 5
4. Is it clear what sources of information were used to compile the publication (other than the author or producer)?	No Partially Yes<br>1 2 3 4 5
5. Is it clear when the information used or reported in the publication was produced?	No Partially Yes<br>1 2 3 4 5
6. Is it balanced and unbiased?	No Partially Yes<br>1 2 3 4 5
7. Does it provide details of additional sources of support and information?	No Partially Yes<br>1 2 3 4 5
8. Does it refer to areas of uncertainty?	No Partially Yes<br>1 2 3 4 5
**Section 2 – How good is the quality of information on treatment choices?**	
9. Does it describe how each treatment works?	No Partially Yes<br>1 2 3 4 5
10. Does it describe the benefits of each treatment?	No Partially Yes<br>1 2 3 4 5
11. Does it describe the risks of each treatment?	No Partially Yes<br>1 2 3 4 5
12. Does it describe what would happen if no treatment is used?	No Partially Yes<br>1 2 3 4 5
13. Does it describe how the treatment choices affect overall quality of life?	No Partially Yes<br>1 2 3 4 5
14. Is it clear that there may be more than one possible treatment choice?	No Partially Yes<br>1 2 3 4 5
15. Does it provide support for share decision-making?	No Partially Yes<br>1 2 3 4 5
**Section 3 – Overall rating of the publication.**	
16. Based on the answers to all of the above questions, rate the overall quality of the publication as a source of information about treatment choices.	Low Moderate High<br>1 2 3 4 5

Section 3
Rating: Low = Serious or extensive shortcomings; Moderate = Potentially important, but not serious shortcomings; High = Minimal shortcomings
