# Supplementary material for: The reliability, functional quality, understandability, and actionability of fall prevention content in YouTube: an observational study
Source: BMC Geriatr. 2022 Aug 9;22:654. doi: 10.1186/s12877-022-03330-x (PMC9362965; doi:10.1186/s12877-022-03330-x)
Supplement: Supplementary file 3 — Additional file 3. [file 12877_2022_3330_MOESM3_ESM.pdf]

Patient Education Materials Assessment Tool for Audiovisual Materials (PEMAT-A/V)

How To Use the PEMAT To Assess a Material

There are seven steps to using the PEMAT to assess a patient education material. The instructions below assume that you will score the PEMAT using paper and pen. If you use the **PEMAT Auto-Scoring Form**, a form that will automatically calculate PEMAT scores once you enter your ratings, you can skip Step 5. The form is available at: http://www.ahrq.gov/professionals/prevention-chronic-care/improve/self-mgmt/pemat/index.html.

Step 1: **Read through the PEMAT and User's Guide.** Before using the PEMAT, read through the entire User's Guide and instrument to familiarize yourself with all the items. In the User's Guide a (P) and (A/V) are listed after an item to indicate whether it is relevant for print and audiovisual materials, respectively.

Step 2: Read or view patient education material. Read through or view the patient education material that you are rating in its entirety.

Step 3: Decide which PEMAT to use. Choose the PEMAT-P for printable materials or the PEMAT-A/V for audiovisual materials.

Step 4: Go through each PEMAT item one by one. All items will have the answer options "Disagree" or "Agree." Some—but not all—items will also have a "Not Applicable" answer option. Go one by one through each of the items, 24 for printable materials and 17 for audiovisual materials, and indicate if you agree or disagree that the material is meeting a specific criterion. Or, when appropriate, select the "Not Applicable" option.

You may refer to the material at any time while you complete the form. You don't have to rely on your memory. Consider each item from a patient perspective. For example, for "Item 1: The material makes its purpose completely evident," ask yourself, "If I were a patient unfamiliar with the subject, would I readily know what the purpose of the material was?"

Step 5: Rate the material on each item as you go. After you determine the rating you would give the material on a specific item, enter the number (or N/A) that corresponds with your answer in the "Rating" column of the PEMAT. Do not score an item as "Not Applicable" unless there is a "Not Applicable" option. Score the material on each item as follows:

If Disagree	Enter 0
If Agree	Enter 1
If Not Applicable	Enter NA

Suggested Citation:

Shoemaker SJ, Wolf MS, Brach C. Patient Education Materials Assessment Tool for Audiovisual Materials (PEMAT-A/V). (Prepared by Abt Associates, under Contract No. HHSA290200900012I, TO 4). Rockville, MD: Agency for Healthcare Research and Quality; October 2013. AHRQ Publication No. 14-0002-EF.

Additional Guidance for Rating the Material on Each Item (Step 5):

- Rate an item "Agree" when a characteristic occurs throughout a material, that is, nearly all of the time (80% to 100%). Your guiding principle is that if there are obvious examples or times when a characteristic could have been met or could have been better met, then the item should be rated "Disagree." The User's Guide provides additional guidance for rating each item.
- Do not skip any items. If there is no "Not Applicable" option, you must score the item 0 (Disagree) or 1 (Agree).
- Do not use any knowledge you have about the subject before you read or view the patient education material. Base your ratings ONLY on what is in the material that you are rating.
- Do not let your rating of one item influence your rating of other items. Be careful to rate each item separately and distinctly from how you rated other items.
- If you are rating more than one material, focus only on the material that you are reviewing and do not try to compare it to the previous material that you looked at.

Step 6: Calculate the material's scores. The PEMAT provides two scores for each material—one for understandability and a separate score for actionability. Make sure you have rated the material on every item, including indicating which items are Not Applicable (N/A). Except for Not Applicable (N/A) items, you will have given each item either 1 point (Agree), or 0 points (Disagree). To score the material, do the following:

- ***Sum the total points*** for the material on the understandability items only.
- ***Divide the sum by the total possible points,*** that is, the number of items on which the material was rated, excluding the items that were scored Not Applicable (N/A).
- ***Multiply the result by 100*** and you will get a percentage (%). This percentage score is the understandability score on the PEMAT.

    - **Example:** If a print material was rated Agree (1 point) on 12 understandability items, Disagree (0 points) on 3 understandability items, and N/A on one understandability item (N/A), the sum would be 12 points out of 15 total possible points (12 + 3, excluding the N/A item). The PEMAT understandability score is 0.8 (12 divided by 15) multiplied by 100 = 80%.

To score the material on actionability, repeat Step 6 for the actionability items.

Step 7: Interpret the PEMAT scores. The higher the score, the more understandable or actionable the material. For example, a material that receives an understandability score of 90% is more understandable than a material that receives an understandability score of 60%, and the same goes for actionability. If you use the PEMAT to rate the understandability and actionability of many materials, you may get a sense of what score indicates exceptionally good or exceptionally poor materials.

Title of Material:

Name of Reviewer: Review Date:

Read the PEMAT User's Guide (available at: http://www.ahrq.gov/professionals/prevention-chronic-care/improve/self-mgmt/pemat/) before rating materials.

UNDERSTANDABILITY

Item #	Item	Response Options	Rating
Topic: **Content**			
**1**	The material makes its purpose completely evident.	Disagree=0, Agree=1	
Topic: **Word Choice & Style**			
**3**	The material uses common, everyday language.	Disagree=0, Agree=1	
**4**	Medical terms are used only to familiarize audience with the terms. When used, medical terms are defined.	Disagree=0, Agree=1	
**5**	The material uses the active voice.	Disagree=0, Agree=1	
Topic: **Organization**			
**8**	The material breaks or "chunks" information into short sections.	Disagree=0, Agree=1, Very short material[1]=N/A	
**9**	The material's sections have informative headers.	Disagree=0, Agree=1, Very short material*=N/A	
**10**	The material presents information in a logical sequence.	Disagree=0, Agree=1	
**11**	The material provides a summary.	Disagree=0, Agree=1, Very short material*=N/A	
Topic: **Layout & Design**			
**12**	The material uses visual cues (e.g., arrows, boxes, bullets, bold, larger font, highlighting) to draw attention to key points.	Disagree=0, Agree=1, Video=N/A	
**13**	Text on the screen is easy to read.	Disagree=0, Agree=1, No text or all text is narrated=N/A	
**14**	The material allows the user to hear the words clearly (e.g., not too fast, not garbled).	Disagree=0, Agree=1, No narration=N/A	

[1] A very short audiovisual material is defined as a video or multimedia presentation that is under 1 minute, or a multimedia material that has 6 or fewer slides or screenshots.

Item #	Item	Response Options	Rating
Topic: **Use of Visual Aids**			
**18**	The material uses illustrations and photographs that are clear and uncluttered.	Disagree=0, Agree=1, No visual aids=N/A	
**19**	The material uses simple tables with short and clear row and column headings.	Disagree=0, Agree=1, No tables=N/A	

***Total Points*: _____________**

***Total Possible Points*: _____________**

Understandability Score (%): _____________
(*Total Points* / *Total Possible Points* × 100)

ACTIONABILITY

Item #	Item	Response Options	Rating
**20**	The material clearly identifies at least one action the user can take.	Disagree=0, Agree=1	
**21**	The material addresses the user directly when describing actions.	Disagree=0, Agree=1	
**22**	The material breaks down any action into manageable, explicit steps.	Disagree=0, Agree=1	
**25**	The material explains how to use the charts, graphs, tables, or diagrams to take actions.	Disagree=0, Agree=1, No charts, graphs, tables, diagrams=N/A	

***Total Points*: _____________**

***Total Possible Points*: _____________**

Actionability Score (%): _____________
(*Total Points* / *Total Possible Points*
