# Supplementary material for: The reliability, functional quality, understandability, and actionability of fall prevention content in YouTube: an observational study
Source: BMC Geriatr. 2022 Aug 9;22:654. doi: 10.1186/s12877-022-03330-x (PMC9362965; doi:10.1186/s12877-022-03330-x)
Supplement: Supplementary file 6 — Additional file 6. [file 12877_2022_3330_MOESM6_ESM.pdf]

Detailed results of the 150-round Wilcoxon rank sum tests

Note:

Identity type 1 = Fitness trainers	Form type 1 = Workout
Identity type 2 = Medical professionals	Form type 2 = Introduction and demonstration
Identity type 3 = Non-professional individuals	Form type 3 = Cartoon
Identity type 4 = Professional organizations	Form type 4 = Monolog
Identity type 5 = Non-professional organizations	Form type 5 = Keynote presentation
p-value < 0.05: significant difference is found	Form type 6 = Drama/Skit

I. The significance between different identities and their video scores.

- Score 1. Overall Quality (DISCERN)

Wilcoxon rank sum test	
W=226.5	p-value=0.0002329

Significance was found between Identity type 1 and 2 in terms of Score 1.

Wilcoxon rank sum test	
W=318	p-value=0.02074

Significance was found between Identity type 1 and 3 in terms of Score 1.

Wilcoxon rank sum test	
W=311.5	p-value=2.358e-05

Significance was found between Identity type 1 and 4 in terms of Score 1.

Wilcoxon rank sum test	
W=186	p-value=0.06136

P > 0.05, no significance was found between Identity type 1 and 5 in terms of Score 1.

Wilcoxon rank sum test	
W=373	p-value=2.785e-05

Significance was found between Identity type 2 and 3 in terms of Score 1.

Wilcoxon rank sum test	
W=619.5	p-value=0.6475

P > 0.05, no significance was found between Identity type 2 and 4 in terms of Score 1.

Wilcoxon rank sum test

W=327.5	p-value=0.09636

P > 0.05, no significance was found between Identity type 2 and 5 in terms of Score 1.

Wilcoxon rank sum test	
W=60.5	p-value=4.219e-06

Significance was found between Identity type 3 and 4 in terms of Score 1.

Wilcoxon rank sum test	
W=37	p-value=0.0008267

Significance was found between Identity type 3 and 5 in terms of Score 1.

Wilcoxon rank sum test	
W=497	p-value=0.04121

Significance was found between Identity type 4 and 5 in terms of Score 1.

- Score 2. Reliability (DISCERN)

Wilcoxon rank sum test	
W=167.5	p-value=1.066e-05

Significance was found between Identity type 1 and 2 in terms of Score 2.

Wilcoxon rank sum test	
W=343.5	p-value=0.004381

Significance was found between Identity type 1 and 3 in terms of Score 2.

Wilcoxon rank sum test	
W=131	p-value=1.611e-09

Significance was found between Identity type 1 and 4 in terms of Score 2.

Wilcoxon rank sum test	
W=311.5	p-value=0.4102

Significance was found between Identity type 1 and 5 in terms of Score 2.

Wilcoxon rank sum test	
W=381.5	p-value=1.586e-05

Significance was found between Identity type 2 and 3 in terms of Score 2.

Wilcoxon rank sum test	
W=604	p-value=0.5405

P > 0.05, no significance was found between Identity type 2 and 4 in terms of Score 2.

Wilcoxon rank sum test	
W=430	p-value=0.0001076

Significance was found between Identity type 2 and 5 in terms of Score 2.

Wilcoxon rank sum test	
W=39	p-value=1.037e-06

Significance was found between Identity type 3 and 4 in terms of Score 2.

Wilcoxon rank sum test	
W=69.5	p-value=0.04994

Significance was found between Identity type 3 and 5 in terms of Score 2.

Wilcoxon rank sum test	
W=674	p-value= 1.397e-06

Significance was found between Identity type 4 and 5 in terms of Score 2.

- Score 3. Functional Quality (DISCERN)

Wilcoxon rank sum test	
W=224	p-value=0.0003077

Significance was found between Identity type 1 and 2 in terms of Score 3.

Wilcoxon rank sum test	
W=316	p-value=0.02813

Significance was found between Identity type 1 and 3 in terms of Score 3.

Wilcoxon rank sum test	
W=267.5	p-value=4.253e-06

Significance was found between Identity type 1 and 4 in terms of Score 3.

Wilcoxon rank sum test	
W=194	p-value=0.1023

P > 0.05, no significance was found between Identity type 1 and 5 in terms of Score 3.

Wilcoxon rank sum test	
W=385	p-value=1.034e-05

Significance was found between Identity type 2 and 3 in terms of Score 3.

Wilcoxon rank sum test	
W=612.5	p-value=0.6038

P > 0.05, no significance was found between Identity type 2 and 4 in terms of Score 3.

Wilcoxon rank sum test	
W=339.5	p-value=0.06161

P > 0.05, no significance was found between Identity type 2 and 5 in terms of Score 3.

Wilcoxon rank sum test	
W=57.5	p-value=5.239e-06

Significance was found between Identity type 3 and 4 in terms of Score 3.

Wilcoxon rank sum test	
W=25	p-value=0.000193

Significance was found between Identity type 3 and 5 in terms of Score 3.

Wilcoxon rank sum test	
W=561	p-value=0.002596

Significance was found between Identity type 4 and 5 in terms of Score 3.

- Score 4. Total Score (DISCERN)

Wilcoxon rank sum test	
W=153.5	p-value=4.271e-06

Significance was found between Identity type 1 and 2 in terms of Score 4.

Wilcoxon rank sum test	
W=369	p-value=0.000548

Significance was found between Identity type 1 and 3 in terms of Score 4.

Wilcoxon rank sum test	
W=163.5	p-value=1.305e-08

Significance was found between Identity type 1 and 4 in terms of Score 4.

Wilcoxon rank sum test	
W=235.5	p-value=0.448

P > 0.05, no significance was found between Identity type 1 and 5 in terms of Score 4.

Wilcoxon rank sum test	
W=404.5	p-value=9.784e-07

Significance was found between Identity type 2 and 3 in terms of Score 4.

Wilcoxon rank sum test	
W=603.5	p-value=0.5372

P > 0.05, no significance was found between Identity type 2 and 4 in terms of Score 4.

Wilcoxon rank sum test	
W=417.5	p-value=0.000329

Significance was found between Identity type 2 and 5 in terms of Score 4.

Wilcoxon rank sum test	
W=32.5	p-value=5.695e-07

Significance was found between Identity type 3 and 4 in terms of Score 4.

Wilcoxon rank sum test	
W=23	p-value=0.0001418

Significance was found between Identity type 3 and 5 in terms of Score 4.

Wilcoxon rank sum test	
W=650	p-value=9.23e-06

Significance was found between Identity type 4 and 5 in terms of Score 4.

- Score 5. Understandability (PEMAT)

Wilcoxon rank sum test	
W=300	p-value=0.01063

Significance was found between Identity type 1 and 2 in terms of Score 5.

Wilcoxon rank sum test	
W=265	p-value=0.3283

P > 0.05, no significance was found between Identity type 1 and 3 in terms of Score 5.

Wilcoxon rank sum test	
W=357	p-value=0.0002243

Significance was found between Identity type 1 and 4 in terms of Score 5.

Wilcoxon rank sum test	
W=273.5	p-value=0.983

P > 0.05, no significance was found between Identity type 1 and 5 in terms of Score 5.

Wilcoxon rank sum test	
W=317	p-value=0.006761

Significance was found between Identity type 2 and 3 in terms of Score 5.

Wilcoxon rank sum test	
W=585.5	p-value=0.4071

P > 0.05, no significance was found between Identity type 2 and 4 in terms of Score 5.

Wilcoxon rank sum test	
W=358	p-value=0.02148

Significance was found between Identity type 2 and 5 in terms of Score 5.

Wilcoxon rank sum test

W=115.5	p-value=0.0004004

Significance was found between Identity type 3 and 4 in terms of Score 5.

Wilcoxon rank sum test	
W=96.5	p-value=0.3763

P > 0.05, no significance was found between Identity type 3 and 5 in terms of Score 5.

Wilcoxon rank sum test	
W=581.5	p-value=0.000698

Significance was found between Identity type 4 and 5 in terms of Score 5.

- Score 6. Actionability (PEMAT)

Wilcoxon rank sum test	
W=610	p-value=0.03927

Significance was found between Identity type 1 and 2 in terms of Score 6.

Wilcoxon rank sum test	
W=362	p-value=0.0002872

Significance was found between Identity type 1 and 3 in terms of Score 6.

Wilcoxon rank sum test	
W=946	p-value=0.003231

Significance was found between Identity type 1 and 4 in terms of Score 6.

Wilcoxon rank sum test	
W=432	p-value=0.0001146

Significance was found between Identity type 1 and 5 in terms of Score 6.

Wilcoxon rank sum test	
W=296	p-value=0.01789

Significance was found between Identity type 2 and 3 in terms of Score 6.

Wilcoxon rank sum test	
W=697.5	p-value=0.6297

P > 0.05, no significance was found between Identity type 2 and 4 in terms of Score 6.

Wilcoxon rank sum test	
W=334.5	p-value=0.03609

Significance was found between Identity type 2 and 5 in terms of Score 6.

Wilcoxon rank sum test	
W=184.5	p-value=0.007739

Significance was found between Identity type 3 and 4 in terms of Score 6.

Wilcoxon rank sum test	
W=95	p-value=0.2418

P > 0.05, no significance was found between Identity type 3 and 5 in terms of Score 6.

Wilcoxon rank sum test	
W=477.5	p-value=0.02705

Significance was found between Identity type 4 and 5 in terms of Score 6.

2. The significance between different forms of expressions and their video scores.

- Score 1. Overall Quality (DISCERN)

Wilcoxon rank sum test	
W=547.5	p-value=0.0005393

Significance was found between form type 1 and 2 in terms of Score 1.

Wilcoxon rank sum test	
W=57	p-value=0.9831

P > 0.05, no significance was found between form type 1 and 3 in terms of Score 1.

Wilcoxon rank sum test	
W=528	p-value=0.7847

P > 0.05, no significance was found between form type 1 and 4 in terms of Score 1.

Wilcoxon rank sum test	
W=169	p-value=0.0005505

Significance was found between form type 1 and 5 in terms of Score 1.

Wilcoxon rank sum test	
W=241.5	p-value=0.1933

P > 0.05, no significance was found between form type 1 and 6 in terms of Score 1.

Wilcoxon rank sum test	
W=45.5	p-value=0.3803

P > 0.05, no significance was found between form type 2 and 3 in terms of Score 1.

Wilcoxon rank sum test	
W=439.5	p-value=0.01439

Significance was found between form type 2 and 4 in terms of Score 1.

Wilcoxon rank sum test	
W=198	p-value=0.433

P > 0.05, no significance was found between form type 2 and 5 in terms of Score 1.

Wilcoxon rank sum test	
W=222	p-value=0.2644

P > 0.05, no significance was found between form type 2 and 6 in terms of Score 1.

Wilcoxon rank sum test	
W=17.5	p-value=0.9009

P > 0.05, no significance was found between form type 3 and 4 in terms of Score 1.

Wilcoxon rank sum test	
W=6.5	p-value=0.2296

P > 0.05, no significance was found between form type 3 and 5 in terms of Score 1.

Wilcoxon rank sum test	
W=9	p-value=0.76

P > 0.05, no significance was found between form type 3 and 6 in terms of Score 1.

Wilcoxon rank sum test	
W=62	p-value=0.007468

Significance was found between form type 4 and 5 in terms of Score 1.

Wilcoxon rank sum test	
W=81	p-value=0.3051

P > 0.05, no significance was found between form type 4 and 6 in terms of Score 1.

Wilcoxon rank sum test	
W=107.5	p-value=0.08639

P > 0.05, no significance was found between form type 5 and 6 in terms of Score 1.

- Score 2. Reliability (DISCERN)

Wilcoxon rank sum test	
W=480	p-value=8.099e-05

Significance was found between form type 1 and 2 in terms of Score 2.

Wilcoxon rank sum test	
W=32.5	p-value=0.3018

P > 0.05, no significance was found between form type 1 and 3 in terms of Score 2.

Wilcoxon rank sum test

W=452	p-value=0.2435

$P > 0.05$, no significance was found between form type 1 and 4 in terms of Score 2.

Wilcoxon rank sum test	
W=134	p-value=0.0001082

Significance was found between form type 1 and 5 in terms of Score 2.

Wilcoxon rank sum test	
W=204.5	p-value=0.06109

$P > 0.05$, no significance was found between form type 1 and 6 in terms of Score 2.

Wilcoxon rank sum test	
W=32.5	p-value=0.9949

$P > 0.05$, no significance was found between form type 2 and 3 in terms of Score 2.

Wilcoxon rank sum test	
W=409.5	p-value=0.06906

$P > 0.05$, no significance was found between form type 2 and 4 in terms of Score 2.

Wilcoxon rank sum test	
W=175.5	p-value=0.1998

$P > 0.05$, no significance was found between form type 2 and 5 in terms of Score 2.

Wilcoxon rank sum test	
W=196.5	p-value=0.6938

$P > 0.05$, no significance was found between form type 2 and 6 in terms of Score 2.

Wilcoxon rank sum test	
W=24	p-value=0.5887

$P > 0.05$, no significance was found between form type 3 and 4 in terms of Score 2.

Wilcoxon rank sum test	
W=13	p-value=0.9365

$P > 0.05$, no significance was found between form type 3 and 5 in terms of Score 2.

Wilcoxon rank sum test	
W=12.5	p-value=0.8433

$P > 0.05$, no significance was found between form type 3 and 6 in terms of Score 2.

Wilcoxon rank sum test	
W=63.5	p-value=0.01178

Significance was found between form type 4 and 5 in terms of Score 2.

Wilcoxon rank sum test	
W=86.5	p-value=0.4508

P > 0.05, no significance was found between form type 4 and 6 in terms of Score 2.

Wilcoxon rank sum test	
W=99	p-value=0.2385

P > 0.05, no significance was found between form type 5 and 6 in terms of Score 2.

- Score 3. Functional Quality (DISCERN)

Wilcoxon rank sum test	
W=364	p-value=9.565e-07

Significance was found between form type 1 and 2 in terms of Score 3.

Wilcoxon rank sum test	
W=58.5	p-value=0.9989

P > 0.05, no significance was found between form type 1 and 3 in terms of Score 3.

Wilcoxon rank sum test	
W=417.5	p-value=0.1151

P > 0.05, no significance was found between form type 1 and 4 in terms of Score 3.

Wilcoxon rank sum test	
W=147.5	p-value=0.0002329

Significance was found between form type 1 and 5 in terms of Score 3.

Wilcoxon rank sum test	
W=185.5	p-value=0.0287

Significance was found between form type 1 and 6 in terms of Score 3.

Wilcoxon rank sum test	
W=54.5	p-value=0.1339

P > 0.05, no significance was found between form type 2 and 3 in terms of Score 3.

Wilcoxon rank sum test	
W=438.5	p-value=0.01744

Significance was found between form type 2 and 4 in terms of Score 3.

Wilcoxon rank sum test	
W=213.5	p-value=0.6918

P > 0.05, no significance was found between form type 2 and 5 in terms of Score 3.

Wilcoxon rank sum test	
W=238.5	p-value=0.1241

P > 0.05, no significance was found between form type 2 and 6 in terms of Score 3.

Wilcoxon rank sum test	
W=14	p-value=0.5866

P > 0.05, no significance was found between form type 3 and 4 in terms of Score 3.

Wilcoxon rank sum test	
W=6	p-value=0.233

P > 0.05, no significance was found between form type 3 and 5 in terms of Score 3.

Wilcoxon rank sum test	
W=7	p-value=0.486

P > 0.05, no significance was found between form type 3 and 6 in terms of Score 3.

Wilcoxon rank sum test	
W=72.5	p-value=0.02822

Significance was found between form type 4 and 5 in terms of Score 3.

Wilcoxon rank sum test	
W=90.5	p-value=0.5589

P > 0.05, no significance was found between form type 4 and 6 in terms of Score 3.

Wilcoxon rank sum test	
W=100	p-value=0.2164

P > 0.05, no significance was found between form type 5 and 6 in terms of Score 3.

- Score 4. Total Score (DISCERN)

Wilcoxon rank sum test	
W=357.5	p-value=7.469e-07

Significance was found between form type 1 and 2 in terms of Score 4.

Wilcoxon rank sum test	
W=51.5	p-value=0.8045

P > 0.05, no significance was found between form type 1 and 3 in terms of Score 4.

Wilcoxon rank sum test	
W=425.5	p-value=0.1392

P > 0.05, no significance was found between form type 1 and 4 in terms of Score 4.

Wilcoxon rank sum test	

W=111	p-value=2.716e-05

Significance was found between form type 1 and 5 in terms of Score 4.

Wilcoxon rank sum test	
W=163.5	p-value=0.01094

Significance was found between form type 1 and 6 in terms of Score 4.

Wilcoxon rank sum test	
W=41.5	p-value=0.5692

P > 0.05, no significance was found between form type 2 and 3 in terms of Score 4.

Wilcoxon rank sum test	
W=435	p-value=0.0213

Significance was found between form type 2 and 4 in terms of Score 4.

Wilcoxon rank sum test	
W=199	p-value=0.4633

P > 0.05, no significance was found between form type 2 and 5 in terms of Score 4.

Wilcoxon rank sum test	
W=220.5	p-value=0.2963

P > 0.05, no significance was found between form type 2 and 6 in terms of Score 4.

Wilcoxon rank sum test	
W=19	p-value=0.9977

P > 0.05, no significance was found between form type 3 and 4 in terms of Score 4.

Wilcoxon rank sum test	
W=8	p-value=0.3822

P > 0.05, no significance was found between form type 3 and 5 in terms of Score 4.

Wilcoxon rank sum test	
W=11	p-value=1.0

P > 0.05, no significance was found between form type 3 and 6 in terms of Score 4.

Wilcoxon rank sum test	
W=64	p-value=0.01248

Significance was found between form type 4 and 5 in terms of Score 4.

Wilcoxon rank sum test	
W=89	p-value=0.5179

P > 0.05, no significance was found between form type 4 and 6 in terms of Score 4.

Wilcoxon rank sum test	
W=103.5	p-value=0.1543

P > 0.05, no significance was found between form type 5 and 6 in terms of Score 4.

- Score 5. Understandability (PEMAT)

Wilcoxon rank sum test	
W=713	p-value=0.04106

Significance was found between form type 1 and 2 in terms of Score 5.

Wilcoxon rank sum test	
W=6	p-value=0.03087

Significance was found between form type 1 and 3 in terms of Score 5.

Wilcoxon rank sum test	
W=332	p-value=0.008895

Significance was found between form type 1 and 4 in terms of Score 5.

Wilcoxon rank sum test	
W=200.5	p-value=0.003086

Significance was found between form type 1 and 5 in terms of Score 5.

Wilcoxon rank sum test	
W=252	p-value=0.2667

P > 0.05, no significance was found between form type 1 and 6 in terms of Score 5.

Wilcoxon rank sum test	
W=9	p-value=0.09055

P > 0.05, no significance was found between form type 2 and 3 in terms of Score 5.

Wilcoxon rank sum test	
W=169.5	p-value=0.4035

P > 0.05, no significance was found between form type 2 and 4 in terms of Score 5.

Wilcoxon rank sum test	
W=172	p-value=0.1675

P > 0.05, no significance was found between form type 2 and 5 in terms of Score 5.

Wilcoxon rank sum test	
W=194	p-value=0.7414

P > 0.05, no significance was found between form type 2 and 6 in terms of Score 5.

Wilcoxon rank sum test

W=32	p-value=0.1325

P > 0.05, no significance was found between form type 3 and 4 in terms of Score 5.

Wilcoxon rank sum test	
W=26	p-value=0.05604

P > 0.05, no significance was found between form type 3 and 5 in terms of Score 5.

Wilcoxon rank sum test	
W=20	p-value=0.08843

P > 0.05, no significance was found between form type 3 and 6 in terms of Score 5.

Wilcoxon rank sum test	
W=111	p-value=0.427

P > 0.05, no significance was found between form type 4 and 5 in terms of Score 5.

Wilcoxon rank sum test	
W=126.5	p-value=0.3515

P > 0.05, no significance was found between form type 4 and 6 in terms of Score 5.

Wilcoxon rank sum test	
W=104	p-value=0.1372

P > 0.05, no significance was found between form type 5 and 6 in terms of Score 5.

6. - Score 6. Actionability (PEMAT)

Wilcoxon rank sum test	
W=1317.5	p-value=0.0006855

Significance was found between form type 1 and 2 in terms of Score 6.

Wilcoxon rank sum test	
W=82.5	p-value=0.2489

P > 0.05, no significance was found between form type 1 and 3 in terms of Score 6.

Wilcoxon rank sum test	
W=905.5	p-value=2.106e-06

Significance was found between form type 1 and 4 in terms of Score 6.

Wilcoxon rank sum test	
W=605.5	p-value=0.001353

Significance was found between form type 1 and 5 in terms of Score 6.

Wilcoxon rank sum test	
W=533.5	p-value=6.821e-05

Significance was found between form type 1 and 6 in terms of Score 6.

Wilcoxon rank sum test	
W=28	p-value=0.6856

P > 0.05, no significance was found between form type 2 and 3 in terms of Score 6.

Wilcoxon rank sum test	
W=405	p-value=0.01508

Significance was found between form type 2 and 4 in terms of Score 6.

Wilcoxon rank sum test	
W=261	p-value=0.3926

P > 0.05, no significance was found between form type 2 and 5 in terms of Score 6.

Wilcoxon rank sum test	
W=245	p-value=0.02397

Significance was found between form type 2 and 6 in terms of Score 6.

Wilcoxon rank sum test	
W=29.5	p-value=0.04892

Significance was found between form type 3 and 4 in terms of Score 6.

Wilcoxon rank sum test	
W=18	p-value=0.5219

P > 0.05, no significance was found between form type 3 and 5 in terms of Score 6.

Wilcoxon rank sum test	
W=17.5	p-value=0.1083

P > 0.05, no significance was found between form type 3 and 6 in terms of Score 6.

Wilcoxon rank sum test	
W=112.5	p-value=0.3074

P > 0.05, no significance was found between form type 4 and 5 in terms of Score 6.

Wilcoxon rank sum test	
W=112.5	p-value=0.5838

P > 0.05, no significance was found between form type 4 and 6 in terms of Score 6.

Wilcoxon rank sum test	
W=93.5	p-value=0.2671

P > 0.05, no significance was found between form type 5 and 6 in terms of Score 6.
